# Comparison of clinical outcomes following delivery of budesonide by both vibrating mesh nebulizer and jet nebulizer in premature infants with bronchopulmonary dysplasia

**DOI:** 10.3389/fped.2023.1258846

**Published:** 2023-11-24

**Authors:** Jian-Fu Zhou, Yi-Bing Zhang, Zhi-Wei Zhang

**Affiliations:** ^1^ Fujian Medical University, Fuzhou, China; ^2^ Department of Neonatology, The Affiliated Hospital of Putian University, Putian, China

**Keywords:** infant, bronchopulmonary dysplasia, vibrating mesh nebulizer, jet nebulizer, high-frequency oscillatory ventilation

## Abstract

**Background:**

This study aimed to compare the efficacy of budesonide inhalation suspension administered via a vibrating mesh nebulizer vs. a jet nebulizer in the treatment of premature infants with bronchopulmonary dysplasia (BPD) undergoing high-frequency oscillatory ventilation (HFOV).

**Methods:**

Between July 2020 and July 2022, we retrospectively analyzed the medical records of 36 preterm infants diagnosed with BPD who underwent HFOV. Based on the nebulizer type used, infants were categorized into the vibrating mesh nebulizer group (VMN group) or the jet nebulizer group (JN group). Post-nebulization outcomes, such as the duration of mechanical ventilation, length of stay in the neonatal intensive care unit (NICU), ventilator-associated parameters, and arterial blood gas metrics, were compared between the two groups. Treatment-associated complications were also documented.

**Results:**

No significant differences were noted between the VMN and JN groups in terms of mechanical ventilation duration (*p *= 0.519), NICU length of stay (*p *= 0.112), ventilator-associated parameters, or complications (*p *= 0.700). However, after 2 weeks of treatment, the oxygenation index (*p *= 0.012) and arterial partial pressure of carbon dioxide (*p *= 0.006) were more favorable in the VMN group compared to the JN group.

**Conclusion:**

Among premature infants with BPD on HFOV, for administration of budesonide inhalation suspension resulted in an improved oxygenation index and reduced arterial partial pressure of carbon dioxide when compared to a jet nebulizer, indicating superior therapeutic efficacy.

## Introduction

With advancements in perinatal medicine and neonatal monitoring technologies, survival rates for premature and low birth weight infants have significantly improved. However, concurrently, we've observed a rise in complications like bronchopulmonary dysplasia (BPD) (1–3). BPD remains a predominant respiratory complication among premature infants, commonly observed in those subjected to prolonged oxygen therapy and/or mechanical ventilation (4, 5). Factors like reduced gestational age, low birth weight, mechanical ventilation, and exposure to high concentrations of inhaled oxygen contribute to lung tissue damage. This damage potentially hampers neonatal lung development and leads to decreased lung function (6–9). Infants diagnosed with BPD often demand extended hospital stays and might encounter respiratory and feeding challenges, cognitive impairments, and risks of cerebral palsy post-discharge. Hence, proactive prevention and treatment become crucial in managing this condition (10). Current therapeutic modalities for BPD include surfactant supplementation, non-invasive or invasive mechanical ventilation, infection prevention, nutritional management, and symptomatic treatments (11–13).

In clinical settings, nebulized glucocorticoids have gained traction due to their efficacious outcomes and controlled dosing. They can swiftly inhibit inflammatory responses, alleviating clinical symptoms in patients (14, 15). Budesonide, with its potent anti-inflammatory properties and lung tissue-targeting capabilities, stands out as a preferred choice (14). Ehrmann's study showed that 99% of respondents working in intensive care units used aerosol therapy during mechanical ventilation, but this finding was not specific to premature infants (15). There's a paucity of research on nebulizer therapy during high-frequency oscillatory ventilation (HFOV), especially in the context of premature infants with BPD. From an aerosol delivery perspective, modeling these patients in bench studies to ascertain metrics like bias flow and drug deposition rate remains challenging. The clinical efficacy can vary significantly based on the aerosol generator device used. Specifically, in neonatal HFOV models, vibrating mesh nebulizers (VMNs) have demonstrated the delivery of inhaled doses over 80 times higher than jet nebulizers (JNs), highlighting a substantial difference in aerosol generation efficiency between these two devices (16). Historically, these devices struggled to accommodate the small tidal volumes, rapid breathing rates, and unfavorable I:E ratios. Thus, this study aims to discern any differences in therapeutic outcomes between the VMN and the JN when used during HFOV in preterm infants diagnosed with BPD.

## Materials and methods

In this retrospective study, we enrolled 36 premature infants diagnosed with BPD who underwent HFOV at our institution between July 2020 and July 2022. The SLE6000 high-frequency oscillatory ventilator (SLE, Croydon, UK) was used for HFOV. All patients' families provided informed consent. The study adhered to the Declaration of Helsinki as set by the World Medical Association and received approval from the hospital's ethics committee.

Inclusion criteria: gestational age ≤32 weeks; immediate admission to the neonatal intensive care unit (NICU) post-birth; underwent HFOV with endotracheal intubation within the first 7 days post-birth; diagnosis consistent with BPD criteria; informed consent from the patient's family. Exclusion criteria: suspected chromosomal abnormalities; significant congenital malformations (e.g., cyanotic congenital heart disease, congenital diaphragmatic hernia); administration of neuromuscular relaxants or history of neuromuscular disease; presence of pulmonary hemorrhage or intracranial hemorrhage upon admission; administration of other types of glucocorticoids during treatment; unilateral discharge or discontinuation of treatment; refusal to participate in the study.

The primary outcome measure was the arterial partial pressure of carbon dioxide (PaCO_2_). Based on literature review findings, the PaCO_2_ for the VMN group was recorded as 44.8, and for the JN group, it was 49.2. With a two-sided α set at 0.05 and a power of 90%, the calculated sample size, using PASS 15.0 software, was *N* = 18 for each group, leading to a total sample size of 36 subjects.

**Figure 1:**
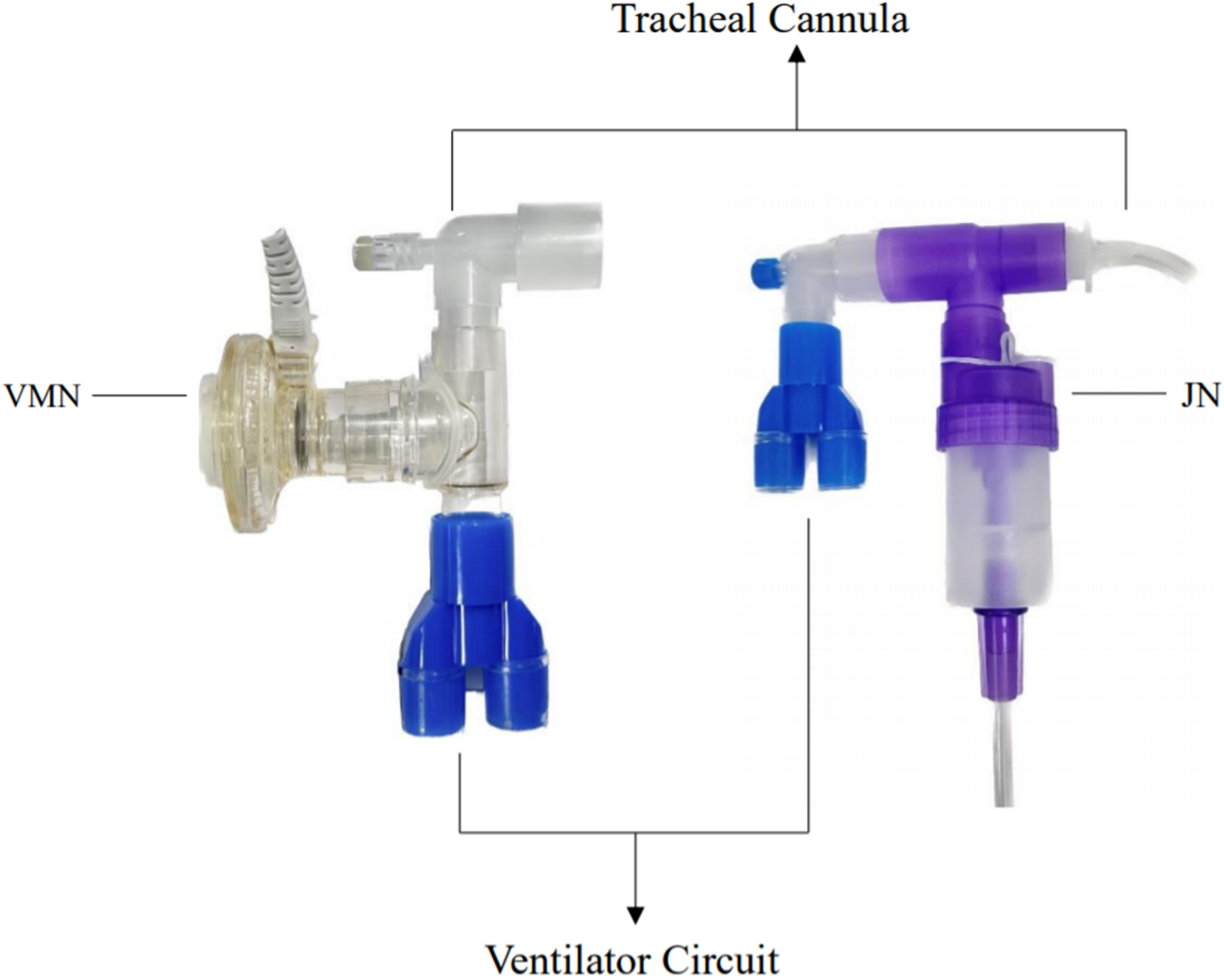
The vibrating mesh nebulizer or jet nebulizer was placed between the endotracheal tube and the ventilator circuit.

From July 2020 to June 2021, 18 infants were treated using a jet nebulizer, designated as the JN group. The jet nebulizer (BaiRui, Inhaler pro compression nebulizer, Shanghai, China) was attached to the ventilator suction line via a T-tube for aerosol administration. From July 2021 to July 2022, another 18 infants received treatment via a vibrating mesh nebulizer, forming the VMN group. This vibrating mesh nebulizer (Aerogen Pro®, in combination with the Aerogen USB controller, Aerogen Ltd., Galway, Ireland) was connected to the ventilator suction line using a curved pipe for aerosol delivery. The nebulizer was placed between the endotracheal tube and the ventilator circuit (Figure 1). Patients were administered 1 ml of budesonide suspension (specification: 2 ml: 1 mg, AstraZeneca Co., Ltd., approval No. LOT325384) for inhalation twice daily, with a total treatment duration of two weeks.

Upon admission, all patients received standard care for preterm infants, encompassing core interventions like thermoregulation, nutritional support, infection prevention, airway patency, and water and electrolyte balance maintenance. Continuous monitoring of vital signs and blood gas analysis was implemented. The same team of NICU physicians and nurses oversaw the care for all patients. Decisions regarding the timing for endotracheal intubation and extubation were at the discretion of the attending NICU physician. Every patient was managed using the SLE 5,000 ventilator (Specialized Laboratory Equipment Ltd., South Croydon, UK) set in the HFOV mode. The ventilator's initial parameters were set as: FiO_2_ ranging from 50% to 100%, frequency between 8 and 12 Hz, an inhalation/exhalation ratio of 1:1, mean airway pressure (MAP) of 10–15 cmH_2_O, and amplitude within 30–40 cmH_2_O. The MAP was increased by 1 cmH_2_O every 2–3 min until there was no further improvement in arterial oxygen saturation. Subsequently, the MAP was decreased by 1–2 cmH_2_O every 2–3 min until a decline in arterial oxygen saturation was noted, then raised again by 1–2 cmH_2_O. Visible chest movement was confirmed by bedside chest x-ray conducted an hour post-HFOV, indicating the right diaphragm's positioning at the 8th–9th posterior rib level. Throughout HFOV, ventilator parameters were adjusted based on arterial blood gas analysis findings. Criteria for extubation included: Stable hemodynamics and adequate spontaneous respiratory function post-discontinuation of sedatives. Ventilator support parameters demonstrating FiO_2_ ≤ 40% and MAP ≤ 8 cmH_2_O. Arterial blood gas (ABG) results indicating PaCO_2_ < 50 mmHg, PaO_2_ > 70 mmHg, pH >7.30, and lactic acid levels below 2.0 mmol/L. Post-extubation, all patients transitioned to nasal continuous positive airway pressure (nCPAP) support.

The primary outcomes of interest were arterial blood gas measurements and the oxygenation index. Arterial blood samples were drawn from all patients both pre-treatment and at 2 weeks post-treatment, and then analyzed using the ABL90 FLEX blood gas analyzer from the USA. Results provided values for the arterial partial pressure of oxygen (PaO_2_) and arterial partial pressure of carbon dioxide (PaCO_2_), from which the oxygenation index (PaO_2_/FiO_2_) was calculated. Secondary outcomes comprised: (1) demographic data: gender, gestational age, birth weight, and the 5-min Apgar score were documented for both groups. (2) ventilator-related parameters: FiO_2_, tidal volume, frequency, and MAP. (3) clinical outcomes: both the duration of mechanical ventilation—from the initiation of endotracheal intubation to extubation—and the total length of NICU stay were tracked. (4) complications: incidence rates of ventilator-associated pneumonia (VAP), pneumothorax, retinopathy of prematurity (ROP), intraventricular hemorrhage (IVH), and necrotizing enterocolitis (NEC) were documented.

For BPD diagnosis, we adhered to the diagnostic criteria and severity classifications put forth by the National Institute of Child Health and Human Development in 2000. BPD was characterized by the need for oxygen concentrations >21% for a minimum of 28 days (17). The severity of BPD was determined either at 36 weeks postmenstrual age or upon discharge: mild BPD required no supplemental oxygen, moderate BPD necessitated <30% oxygen, and severe BPD required ≥30% oxygen and/or positive pressure ventilation. The diagnosis of VAP was grounded in the diagnostic criteria specified for infants under 1 year of age by the U.S. (18). Centers for Disease Control and Prevention. Pneumothorax was identified through bedside chest x-rays. The revised 2005 Classification of Retinopathy of Prematurity was used to diagnose ROP (19). IVH was detected via bedside cranial ultrasounds (20). Lastly, necrotizing enterocolitis was diagnosed based on the modified Bell's staging criteria (21).

## Statistical analysis

Data were processed using SPSS 26.0 software. To determine the normality of data distribution, we carried out a Kolmogorov–Smirnov test before any subsequent analyses. For normally distributed data, *t*-test analyses were utilized; if not, the Wilcoxon-Mann–Whitney test was applied. Qualitative data between both groups were compared using the Chi-square test. When the conditions for the χ^2^ test weren't met, we employed Fisher's exact test. A *P* value less than 0.05 was deemed statistically significant.

## Results

In this study, 36 neonates were included. They were categorized based on the nebulizer type into the VMN group (*n* = 18) and the JN group (*n* = 18). Pre-treatment, the PaO_2_ (*p *= 0.889), PaCO_2_ (*p *= 0.368), and oxygenation index (*p *= 0.170) didn't show significant differences between both groups. However, post two-week treatment, both groups saw significant improvements in PaO_2_ when compared to their initial values. Yet, there wasn't any noticeable difference in post-treatment PaO_2_ between the groups (*p *= 0.388). After the two-week treatment, the VMN group had a notably lower PaCO_2_ (*p *= 0.006) and a higher oxygenation index (*p *= 0.012) compared to the JN group (Table 1).

**Table 1:** Primary outcome.

Items		VMN	JN	*P*-value
Before treatment	PaO_2_	46.5 ± 5.8	45.6 ± 4.9	0.889
	PaCO_2_	44.8 ± 4.1	45.9 ± 3.1	0.368
	PaO_2_/FiO_2_	166.7 ± 37.3	187.0 ± 48.7	0.170
After 2 weeks of treatment	PaO_2_	81.1 ± 9.4	78.8 ± 5.3	0.388
	PaCO_2_	37.1 ± 3.0	40.5 ± 4.1	0.006
	PaO_2_/FiO_2_	331.9 ± 49.9	290.7 ± 43.6	0.012

The gender (*p *= 0.738), gestational age (*p *= 0.213), birth weight (*p *= 0.138), and 5-min Apgar scores (*p *= 0.074) were similar between the two groups, ensuring the general characteristics of the cohorts were comparable (Table 2). When considering ventilator-related metrics like FiO_2_, tidal volume, frequency, and MAP, no significant difference was found between the two groups both before and after the two-week treatment (Table 3). Additionally, the duration of mechanical ventilation (*p *= 0.519) and the length of the NICU stay (*p *= 0.112) did not significantly differ between the two groups (Table 4).

**Table 2:** Comparison of general data.

Items	VMN	JN	*P*-value
Number of patients (*n*)	18	18	
Gender (M/F)	10/8	9/9	0.738
Gestational age (weeks)	30.4 ± 3.2	29.1 ± 2.8	0.213
Birth weight (g)	1,204 ± 174.9	1,282 ± 130.0	0.138
Apgar score at 5 min of birth	6.2 ± 1.8	5.1 ± 1.8	0.074

**Table 3:** Comparison of ventilator related parameters.

Items		VMN	JN	*P*-value
Before treatment	FiO_2_ (%)	100 (100,100)	100 (100,100)	0.981
	Tidal volume (ml)	19.6 ± 3.6	20.3 ± 2.7	0.472
	Frequency (Hz)	15.0 ± 1.9	15.9 ± 2.2	0.185
	MAP (cmH_2_O)	14.7 ± 2.7	15.4 ± 2.6	0.457
After 2 weeks of treatment	FiO_2_ (%)	100 (95,100)	100 (96.5,100)	0.470
	Tidal volume (ml)	22.8 ± 3.8	24.2 ± 2.8	0.206
	Frequency (Hz)	13.6 ± 1.9	14.8 ± 3.2	0.152
	MAP (cmH_2_O)	12.3 ± 2.1	11.8 ± 3.2	0.547

**Table 4:** Comparison of other outcomes.

Items	VMN	JN	*P*-value
Duration of mechanical ventilation (*d*)	15.1 ± 3.2	15.8 ± 2.5	0.519
Length of NICU stay (*d*)	20.2 ± 5.2	22.6 ± 3.7	0.112
Complications [*n* (%)]	5 (27.8)	4 (22.2)	0.700
VAP (*n*)	1	0	
ROP (*n*)	1	2	
NEC (*n*)	2	1	
IVH (*n*)	0	1	
Pneumothorax (*n*)	1	0	

NICU, neonatal intensive care unit; VAP, ventilator-associated pneumonia; ROP, retinopathy of prematurity; NEC, necrotizing enterocolitis; IVH, intraventricular hemorrhage.

## Discussion

There is an inverse relationship between the occurrence of BPD and clinical parameters like gestational age and birth weight. Ultra-premature infants (those with a gestational age below 28 weeks) exhibit BPD incidence rates ranging from 48%–68% (22, 23). For preterm infants weighing between 500 and 1,500 g, the BPD incidence is approximately 22%, with those weighing between 500 and 750 g experiencing a staggering 42% incidence (24, 25). Our study encompassed 36 preterm infants with gestational ages spanning 23–35 weeks and weights ranging from 0.89–2.81 kg. Predominant causes leading to preterm births among these infants included maternal gestational diabetes mellitus, hypertension, and premature rupture of membranes, among others. Post-birth, all these infants necessitated endotracheal intubation and mechanical ventilation, exhibiting varying degrees of oxygen dependency. Based on the BPD diagnostic and severity criteria, all participants in this study were categorized as having severe BPD.

Glucocorticoids, due to their potent anti-inflammatory and immunomodulatory properties, have become a staple in BPD prevention and treatment. These properties allow glucocorticoids to minimize pulmonary edema, decrease the physiological demand for oxygen, and abbreviate the duration of mechanical ventilation (26, 27). The administration of glucocorticoid via aerosol inhalation, targeting the airway mucosa directly, accentuates its localized respiratory effects. Notably, it substantially curtails BPD incidence and the duration of oxygen utilization (28). Several factors can influence the efficacy of nebulization, with the choice of drug being paramount. For consistency, this study solely used budesonide suspension for aerosol inhalation in all participating infants. Previous research has consistently shown that budesonide is effective in preventing and treating BPD in premature infants (14, 29, 30). It not only reduces the incidence and complications of BPD but also improves blood gas metrics, curtails inflammation, and enhances pulmonary function in these infants. By exclusively using budesonide for nebulization, this study aimed to optimize drug-related efficacy while reducing potential confounding factors. Using a standardized therapeutic agent enabled a clearer evaluation of the nebulization's impact on patient outcomes.

The Aerogen Pro electronic nebulizer employs a sophisticated micropump system to produce precise aerosol generation. Its OnQTM aerosol generator features a vibrating membrane punctured by roughly 1,000 tapered apertures. When vibrated at ultrasonic frequencies, fluid is forced through these openings, creating controlled streams that fragment into optimized droplets. The diameter of the holes meticulously adjusts droplet size, yielding tailored particle distributions with minimal residual volumes in the microliter range. On the other hand, jet nebulizers, frequently used for aerosol delivery in intubated neonates, have shown inefficiencies. Research has highlighted notable performance disparities between different jet nebulizer brands (31). Inefficiencies largely arise from hygroscopic growth, where particles expand due to interactions with moist gas, and from drug losses in the expiratory limb during continuous flow. Furthermore, the narrow endotracheal tubes used in newborns present significant challenges to effective drug deposition.

Following two weeks of nebulizer treatment, there was a marked decrement in PaCO_2_ levels across both groups. The magnitude of PaCO_2_ decline in the VMN group surpassed that of the JN group. Concurrently, the oxygenation index was substantially elevated in the VMN group compared to the JN cohort. This insinuates that pulmonary ventilation function enhancement was more pronounced in the VMN group, thus facilitating more efficient carbon dioxide excretion. The enhanced performance of the vibrating mesh nebulizer may be attributed to its ability to produce a consistent, uniform aerosol with an average particle diameter ranging from 1–5 μm. This ensures efficient drug deposition deep into the respiratory tree, especially in the distal 5–6 airways, maximizing drug delivery efficacy (32). Numerous *in vitro* investigations have corroborated that the vibrating mesh nebulizers outperform both jet and ultrasonic nebulizers in terms of aerosol drug delivery rate and lung deposition (33–35). Dubus et al.'s study on neonatal mechanically ventilated rhesus monkeys unveiled that lung deposition rate using the vibrating mesh nebulizer was an astounding 20-fold higher than with jet nebulizers (36). In our study, to eliminate potential variations from nebulizer placement, both the vibrating mesh and jet nebulizers were strategically positioned between the endotracheal tube and the ventilator circuit.

Pre-treatment comparative analysis in our study revealed no significant disparities between the two patient groups in terms of general data, initial therapeutic regimes, and requisite ventilator parameters. Furthermore, baseline ventilator parameters (encompassing FiO_2_, tidal volume, frequency, and MAP) demonstrated equivalence across the cohorts. Post two-week nebulizer intervention, there was a noticeable decline in the ventilator parameters for both groups. This decline, albeit more pronounced in the VMN group than the JN group, did not reach statistical significance. Yet, the data suggests a reduced dependence on ventilator pressure and oxygen in the VMN cohort. The implications of these findings underscore the potential benefits of the VMN group and beckon larger sample, extended duration studies in the future.

The duration of mechanical ventilation and the length of NICU stay in the VMN group were slightly shorter than those in the JN group, but the difference was not statistically significant. Mechanical ventilation is essential for the prevention and treatment of BPD in premature infants. However, its use can increase the risk of respiratory tract infection and potential brain tissue injury. A ventilator-associated infection, like VAP, can worsen lung function in premature infants within a year. In our study, the incidence of VAP between the two groups was low, with no significant difference. Additionally, the use of a vibrating mesh nebulizer did not elevate the risk of complications such as pneumothorax, ROP, IVH, and NEC. This is consistent with findings from Dugernier and Pitance et al. (37, 38).

A significant novel finding from our study is the evident superior clinical outcomes using the vibrating mesh nebulizer for budesonide aerosol delivery compared to jet nebulizer administration in patients with bronchopulmonary dysplasia. To the best of our knowledge, this represents the first direct evidence of improved patient outcomes with VMN-administered budesonide therapy. We theorize that the VMN's markedly increased aerosol output and inhaled dose delivery are foundational to its therapeutic advantages—a hypothesis aligned with bench studies that show an over 80-fold higher dose with VMN in mechanical ventilation settings. While previous data hinted at the superior efficacy of VMN, our research distinctively underscores the translational potential of VMN aerosol generators in enhancing clinical responses compared to jet nebulizers.

In addition, previous studies indicated that vibrating mesh nebulizers were more effective in treating children with asthma and severe pneumonia (31, 39). DiBlasi et al. reached a similar conclusion in a vitro study using an infant model (40). Future studies should investigate if these findings apply to other respiratory conditions. VMN might offer increased advantages for patients who need extended mechanical ventilation support, like infants with ARDS. Prior studies on VMN have incorporated *in vitro* models, rhesus monkey models, and infants. Future research could further explore the use of VMN in children.

However, it's essential to acknowledge the limitations inherent in our study. Being a retrospective investigation from a single center, it lacked randomization and involved a restricted patient cohort. We recognize the need for larger, multi-center, randomized studies to further validate and enhance the generalizability of our findings. Furthermore, the uniform dosage of budesonide across both groups, without accounting for possible variances in drug delivery due to the distinct inhalation mechanisms, might have influenced our results. Additionally, keeping the ventilation parameters consistent during nebulization might have influenced pulmonary drug deposition and the effects of treatment. Lastly, while our study shed light on short-term outcomes associated with the vibrating mesh nebulizer, the long-term clinical ramifications remain to be elucidated.

## Conclusions

In sum, for premature infants with BPD on HFOV, the employment of a vibrating mesh nebulizer for budesonide inhalation suspension, relative to a jet nebulizer, seems to offer tangible therapeutic benefits by effectively lowering PaCO_2_, enhancing the oxygenation index, and thus presenting a preferable therapeutic strategy.

## Data Availability

The raw data supporting the conclusions of this article will be made available by the authors, without undue reservation.
